# Change in active transportation and weight gain in pregnancy

**DOI:** 10.1186/s12966-016-0332-7

**Published:** 2016-01-27

**Authors:** Marianne Skreden, Nina C. Øverby, Linda R. Sagedal, Ingvild Vistad, Monica K. Torstveit, Hilde Lohne-Seiler, Elling Bere

**Affiliations:** Department of Public Health, Sports and Nutrition, University of Agder, Kristiansand, Norway; Department of Obstetrics and Gynaecology, Sørlandet Hospital, Kristiansand, Norway

**Keywords:** Active transportation, Biking, BMI, Pregnancy, Public transportation, Walking, Weight gain

## Abstract

**Background:**

Pregnancy is characterised by large weight gain over a short period, and often a notable change in mode of transportation. This makes pregnancy suitable for examining the plausible, but in the scientific literature still unclear, association between active transportation and weight gain. We hypothesize that women continuing an active mode of transportation to work or school from pre- to early pregnancy will have a lower gestational weight gain (GWG) than those who change to a less active mode of transportation.

**Methods:**

We analysed prospective data from the Norwegian Fit for Delivery (NFFD) trial. Between September 2009 and February 2013 606 women were consecutively enrolled in median gestational week 16 (range; 8–20). Of 219 women who used an active mode of transportation (biking, walking, public transportation) pre-pregnancy, 66 (30 %) converted to a less active mode in early pregnancy (“active-less active” group), and 153 (70 %) continued with active transportation (“active-active” group). Pre-pregnancy weight was self-reported. Weight at gestational (GA) weeks 16, 30, 36, and at term delivery was objectively measured. Weight gain was compared between the two groups. Linear mixed effects analysis of the repeated weight measures was performed including the group*time interaction.

**Results:**

A significant overall group effect was observed for the four time points together (“active-active” group: 77.3 kg vs. “active-less active” group: 78.8 kg, *p* = 0.008). The interaction term group*time was significant indicating different weight gain throughout pregnancy for the two groups; the mean differences between the groups were 0.7 kg at week 16, 1.4 kg at week 30, 2.1 kg at week 36, and 2.2 kg at term delivery, respectively.

**Conclusion:**

The findings indicate that active transportation is one possible approach to prevent excessive weight gain in pregnancy.

## Background

In the last few decades overweight and obesity have become a major threat to public health worldwide [1]. Preventing weight gain is preferable to treating overweight and obesity because of the limited sustainability of weight loss [2]. Gestational weight gain (GWG) in line with the recommendations given by the Institute of Medicine [3], is known to optimize mother and child health outcome [3]. However, appropriate GWG is achieved by a minority of pregnant women in most parts of the world [4, 5].

In general physical activity is inversely associated with weight gain [6–8], and increasing physical activity is a priority [9, 10]. Although there are few contraindications to low to moderate intensive physical activity in pregnancy, reduction in physical activity level is the norm compared to pre-pregnancy [11–17]. Common barriers to physical activity during pregnancy are lack of time and pregnancy symptoms [12, 15, 18–20]. Active transportation is one way to promote physical activity in pregnancy, and has also environmental benefits [21]. Furthermore, active transportation is often built into people’s day-to day routines, facilitating adherence [22]. The literature on active transportation to school or work in pregnancy is scarce. Private car seems to be the most frequent mode of transportation among pregnant women in developed countries [23–25]. We have earlier reported that 66 % used private transport, 10 % walked, 8 % biked and 16 % used public transportation to work or school before GA week 20 [24]. Haakstad et al. found that 53 % drove their own car, while 32 % used public transport and 15 % walked or biked to work in third trimester of pregnancy [25]. Pregnant women bike less in pregnancy, compared to pre-pregnancy [12, 25], and they often continue walking [12, 25].

Both longitudinal [26], and large population-based studies [27–29] in non-pregnant populations have reported that active transportation to work was associated with lower BMI [27, 28], and lower probability of being overweight [29]. A similar association has been demonstrated in longitudinal studies among children [30] and adolecents [31] and among adults in two recent systematic reviews [32, 33]. Most studies included in these two systematic reviews were, however, cross-sectional. Furthermore, heterogenous study design and lack of comparability of outcomes weakened the conclusions. A third systematic review concluded that there was little robust evidence that active transportation intervention prevents obesity [34]. Randomised controlled intervention trials in non-pregnant populations have reported mixed findings on the association between active transportation and reduction in body weight [35–37]. In order to detect significant associations between active transportation and weight gain, large-scale randomized trials manipulating mode of transportation over a long time period are needed. Such trials would, however, be hard to conduct and probably not feasible. Because of these shortcomings, there has been a call for longitudinal and more explorative studies in adults [27, 32–34].

Pregnancy is characterized by a series of specific metabolic changes such as the stimulation of accretion of adipose tissue in early pregnancy and increased insulin resistance later in pregnancy [38]. Furthermore, there is a notable change in the mode of transportation from pre-pregnancy to early pregnancy. Thus, we hypothesize that women continuing an active mode of transportation to work or school from pre-pregnancy to early pregnancy (≤20 GA weeks) will have lower GWG than those who change to a less active mode of transportation.

## Methods

We analysed prospective data from the Norwegian Fit for Delivery (NFFD) trial, where half of the participants were randomised to antenatal nutritional counselling and a physical activity program. The main aims of the NFFD trial were to examine the effect on maternal GWG, newborn birth weight, glucose regulation during pregnancy, complications of pregnancy and delivery, and postpartum maternal weight retention. The protocol for the NFFD trial has previously been described in detail [39].

Between September 2009 and February 2013 pregnant nulliparous women were consecutively recruited from eight local antenatal clinics around Kristiansand in southern Norway. Participants provided informed consent at inclusion. Additional inclusion criteria were singleton pregnancy, GA ≤ 20 weeks, BMI ≥ 19.0 kg/m^2^, and literacy in Norwegian or English. Women with diabetes mellitus, ongoing substance abuse, physical disability which precluded participation in a physical activity program, or planned relocation outside the study area before delivery were ineligible. For the present study women who did not work or study before pregnancy or at inclusion and women who used private transportation to work before pregnancy were excluded.

The Norwegian Regional Committee for Medical Research Ethics South-East C approved the trial and modifications (REK reference 2009/429). The trial was registered prospectively in the ClinicalTrials.gov database, NCT01001689.

### Weight measures

At inclusion, in median GA week 16.0 (range; 8.0–20.0), the women reported their pre-pregnancy weight and were weighed at their local health care clinic. The participants’ height was measured using a Seca Leicester stadiometer at GA week 30. Weight was measured at Sorlandet hospital at GA weeks 30 and 36 using a Tanita bioimpedance scale. Furthermore, the women were weighed on admission to the delivery ward. If missing, weight recorded in the antenatal record within two weeks of admission was allowed. Pre-pregnant self-reported weight was used for calculation of pre-pregnancy BMI (weight/height^2^).

### Assessment of mode of transportation to work or school

At inclusion, the women answered the questions “How do you usually get to work/school now?” and “How did you usually get to work/school before pregnancy?” Both questions had six response alternatives: “walk”, “bike”, “public transportation (bus, train, etc.)”, “car”, “motorcycle, scooter or moped”, and “not applicable (not working, going to school)”. Participants were only able to select one response alternative. Thus information on multi-mode trips was not captured.

Test-retest properties were assessed with data from a previous study [40] where pregnant women reported pre-pregnant and current mode of transportation to work or school 14 days apart. The test-retest agreement was 95 % (88 out of 93) (kappa measure of agreement 0.80) and 89 % (85 out of 95) (kappa measure of agreement 0.73) for pre-pregnant and current mode of transportation to work or school, respectively.

“Walking” and “biking” were categorized as “active” transportation. Public transportation was also categorized as “active” transportation [41], as it is documented that persons who use public transportation often bike or walk to and from bus stops and train stations [42, 43].

In the current study, we examined the effect of the behaviour of changing to *less active* mode of transportation from pre-pregnancy to early pregnancy. Women who changed to a *less* active mode of transportation, i.e., women who changed from “walk” or “bike” to “public transportation” and women who changed from “walk” or “bike” or “public transportation” to “car” were categorized as “active-less active”. Women who used the same mode of transportation in early pregnancy as pre-pregnancy and women who changed mode of transportation from “walk” to “bike” or “bike” to “walk” were categorized as “active-active”.

### Other study variables

Information on maternal age at inclusion, educational level, pre-pregnancy smoking, pre-pregnancy diet quality, pre-pregnancy physical activity levels, and early pregnancy symptoms were obtained from the questionnaire filled in at inclusion. Data on length of gestation at delivery and preeclampsia were extracted from the hospital medical chart. The women reported their level of education by choosing one of the following response options: ‘less than 7 years of primary education’, ‘7–10 years of primary education’, ‘trade school or 1–2 years of high school’, ‘completed high school’, ‘less than 4 years at college/university’ and ‘4 years or more at college/university’. Educational level was dichotomized into low education (did not attend college or university) and high education (did attend college or university). Pre-pregnant smoking had the following response options: ‘never smoked’, ‘smoked before I became pregnant, but have stopped completely’, ‘smoke 1–4 cigs/day’, ‘smoke 5–9 cigs/day’, ‘smoke 10–20 cigs/day’ and ‘smoke > 20 cigs / day’. Smoking was collapsed into ‘nonsmoker’ and ‘current/previous smoker’. Pre-pregnant diet quality (NFFD-diet score) was assessed by a 43 item Food Frequency Questionnaire at inclusion [40]. Pre-pregnant physical activity levels were assessed with the International Physical Activity Questionnaire short version (IPAQ-S) [44]. IPAQ-S assesses physical activity level in all arenas (leisure-time, occupation, house-hold and transport) the last seven days. MET (Metabolic Equivalent Task) score (MET-minutes*week^-1^) was calculated as outlined in the IPAQ manual [44]. At inclusion the women were asked about the most important reasons for not doing physical activities. One possible answer was ‘because of nausea’. We categorized this into ‘having nausea’ vs. ‘not having nausea’. We defined preeclampsia using the diagnostic criteria for preeclampsia defined by the Norwegian Federation of Obstetricians and Gynaecologists [45] and categorized findings as ‘preeclampsia’ vs. ‘not preeclampsia’. Preterm birth was defined as delivery between 22 and 37 weeks of gestation.

### Statistical methods

The analyses were performed with IBM SPSS statistics 22 (IBM Corporation, Armonk, NY, USA). A two-sided p value of 0.05 was considered significant. For comparison of unpaired categorical and continuous data, we used Pearson’s *χ*^2^ test and independent sample *t*-test, respectively. Linear mixed effects analyses of repeated measures (i.e., at inclusion (gestational week 16), gestational week 30, gestational week 36 and at term delivery) was performed for maternal weight gain. The models were estimated using an unstructured covariance matrix. As fixed effects, the model included maternal age, maternal pre-pregnancy BMI, maternal height, educational level, pre-pregnancy smoking, pre-pregnancy NFFD-diet score, pre-pregnancy physical activity, nausea at inclusion, preeclampsia, gestational length at delivery, and randomisation into the NFFD trial. The total effect of being in the “active-less active” group was defined as the differences in maternal weight gain between “active-less active” and “active- active” group at follow-up at GA weeks, 16, 30, 36, and term delivery. The interaction term group*time was included as a measure of differences in the effect over time for the “active-active” group vs. the “active-less active” group. A significant interaction was defined as *p* value < 0.10 for the product term.

## Results

**Fig. 1:**
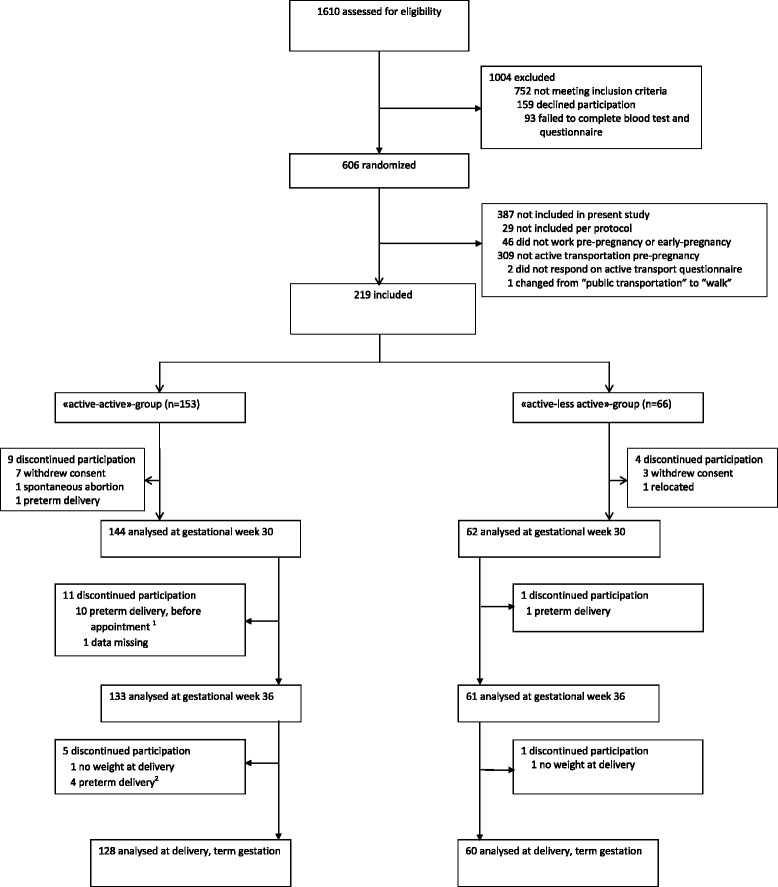
Inclusion and exclusion of women through the study period. ^1^number in “active-active” group who delivered before gestational (GA)week 36 is 11, but there is a slight discordance in attendance at week 36 measurements because of variations in timing of appointment (34–38 weeks gestation). ^2^number in “active-active” group who delivered after GA week 36 but before GA week 37 is 3

The recruitment of 606 pregnant women into the NFFD trial is shown in Fig. 1. Twenty nine were excluded per protocol and 309 used cars or other motorized vehicles as mode of transportation to work or school pre-pregnancy. Furthermore, 46 women did not work before they got pregnant or at inclusion, two women did not answer the questions on active transportation, and one woman changed to a more active mode of transportation (e.g., from “public transportation” to “walk”). Therefore 219 women were included in the final analysis. Comparisons between the 219 included women and the 358 women who were excluded from the present study, showed that included women reported more favourable pre-pregnancy NFFD-diet score (*p* = 0.003), less often smoked pre-pregnancy (*p* = 0.01) and had more frequent preeclampsia (*p* = 0.038) than excluded women. There were no statistically significant differences between the included and excluded women in age, height, educational level, gestational age at inclusion, reporting nausea as reason for not being physically active at inclusion or pre-pregnant weight, BMI or, physical activity level (Table 1).

**Table 1:** Maternal characteristics

	All (*n* = 219)	Active-less active (*n* = 66)	Active-active (*n* = 153)	*p* value^*^
NFFD intervention group^†^	49.8 (109)	47.0 (31)	51.0 (78)	0.586
Maternal age (years)^‡^	27.8 (4.4)	28.3 (4.5)	27.6 (4.3)	0.243
Height (cm)^¥^	168.5 (6.0)	169.0 (6.1)	168.3 (5.9)	0.463
Weight (kg)^¥^	67.4 (11.7)	68.2 (12.9)	67.1 (11.2)	0.508
BMI (kg/cm^2^)^¥^	23.7 (3.8)	23.8 (4.0)	23.7 (3.7)	0.747
Not college education	28.8 (63)	24.2 (16)	30.7 (47)	0.331
Non-smoker^¥^	74.2 (167)	76.1 (51)	73.4 (116)	0.672
NFFD-diet score^¥^	4.9 (2.2)	4.6 (2.4)	5.0 (2.2)	0.212
MET score (MET-minutes*week^-1^)^¥^	2396 (1879)	2353 (1726)	2415 (1949)	0.824
MET score (MET-minutes*week^-1^)^‡^	1649 (1497)^a^	1428 (1101)^c^	1743 (1643)^b^	0.192
∆ MET score (MET-minutes*week^-1^)	835 (1241)^a^	943 (1341)^c^	790 (1199)^b^	0.445
Nausea^‡^	30.7 (69)	32.8 (22)	26.6 (42)	0.646
Preeclampsia	6.6 (14)^d^	6.3 (4)^f^	6.8(10)^e^	1.0
Gestational age at delivery (days)	279 (14)	282 (12)	278 (15)	0.039

Data are mean (SD) and % (n). Active-active group; Changed from “walk/bike to walk/bike”, or from “public transportation to public transportation”

Active-less active group; Changed from “walk/bike to public transportation/car”, or changed from “public transportation” to “car”

*BMI* Body Mass Index, *MET* Metabolic Equivalent Task, *NFFD* Norwegian fit for delivery

^*^ *p* value; based on comparison between “Active-active” and “Active-less active” groups. ^†^ Randomised to intervention group in the NFFD trial. ^‡^ At inclusion (median gestational weeks 16.0 (range 8.0–20.0)). ^¥^ Pre-pregnancy. ^a^ *n* = 184, ^b^ *n* = 129, ^c^ *n* = 55, ^d^ *n* = 212, ^e^ *n* = 148, ^f^ *n* = 64

In early pregnancy, 66 (30 %) of the 219 included women had changed to a less active mode of transportation (“active-less active”), and 153 (70 %) continued with active transportation to work or school (“active-active”). Baseline characteristics for the “active-less active” and the “active-active” groups are shown in Table 1. There were no significant differences in the two groups regarding maternal age, educational level, nausea at inclusion, preeclampsia, pre-pregnant weight, BMI, smoking, NFFD-diet score or physical activity level (Table 1). Gestational age at delivery was significant longer in the “active-less active” group than the “active-active” group (*p* = 0.039). The prevalence of premature delivery (GA < 37 weeks) was 1.6 % and 10.5 % (*p* = 0.026) in the “active-less active” group and the “active-active” group, respectively.

Significant effect of continuing active transportation in early pregnancy was observed for the four timepoints together on maternal weight; 77.3 kg v. 78.8 kg (*p* = 0.008) (Table 2). Since the interaction term group*time was significant at 0.1 level (*p* = 0.073), weight gain through pregnancy was significantly different between the “active-active” group and the “active-less active” group. The “active-less active” group had an increasingly higher mean GWG through pregnancy compared to the “active-active” group); mean difference in GWG between the groups was 0.7 kg at week 16, 1.4 kg at week 30, 2.1 kg at week 36, and 2.2 kg at term delivery, respectively (Table 2).

**Table 2:** Maternal weight (kg) through pregnancy

	Active-less active (*n* = 66)	Active-active (*n* = 153)	Difference ^a^
Overall weight (all time points)	78.8 (77.6–80.1)	77.3 (76.2–78.3)	1.5^b^
Weight week 16	70.9 (69.9–72.0)	70.2 (69.3–71.2)	0.7
Weight week 30	78.2 (76.9–79.5)	76.8 (75.7–77.8)	1.4
Weight week 36	82.0 (80.6–83.4)	79.9 (78.8–81.1)	2.1
Weight at term delivery	84.3 (82.7–85.8)	82.1 (80.9–83.3)	2.2

Data presented as mean (95 % CI)

^a^ Difference in weight between “active-less active” group” and “active-active” group. Multilevel repeated linear mixed model, adjusting for maternal age, pre-pregnancy BMI, maternal height, educational level, pre-pregnancy Norwegian fit for delivery (NFFD)-diet score, pre-pregnancy physical activity, pre-pregnancy smoking, nausea, gestational length at delivery and being randomised to the intervention group in the NFFD trial

^b^ overall group effect was observed for the four time points together (*p* = 0.008)

## Discussion

We have compared GWG in women who continued with active transportation in early pregnancy with women who converted from active transportation to more passive transportation to work or school. We found that women who continued active transportation to work or school from pre- to early pregnancy had a significantly lower weight gain through pregnancy compared to women who changed to a less active mode of transportation. Other studies have also reported that physical activity in pregnancy is inversely associated with GWG [4, 25]. The results from the present study are in line with a recent longitudinal and three large population-based cross-sectional studies all done in non-pregnant populations, reporting a dose dependant reduction in BMI with active transportation to work [26, 28] and a lower likelihood of overweight and obesity [27, 29]. A longitudinal Australian study reported an association between daily travel to work and weight gain [46], and a cross sectional study reported an association between walking to work and lower BMI, while biking to work was associated with lower BMI in men, but not in women [47]. Similarly, a longitudinal study among Norwegian and Dutch adolescents showed that those who stopped biking to school had higher odds of being overweight than those who continued biking [31]. The three randomized trials done on active transportation and weight were done in non-pregnant populations [35–37]. In a small Danish randomised controlled trial, the commuter biking group had a significant reduction in skinfold thickness after eight weeks compared to the non-commuter biking group [37], while other randomized trials did not find any effect of biking to work on weight [35, 36].

Identifying barriers to active transportation in pregnancy is important, as pregnancy has been associated with a sharp decline in frequency and amount of physical activity [11–13, 15–17]. Pregnancy is recognized as a period of transition with social, biological, psychological and behavioural change. Studies on traditional beliefs connected to lifestyle in pregnancy have generally been conducted in indigenous populations [48, 49] or in ethnic minorities in developed countries [50]. Most of these studies have reported that pregnancy is viewed as a vulnerable time which requires rest and protection, and women are advised to reduced their physical activity level [48–50]. Traditional beliefs on these topics are less explored in western societies. Several pregnancy specific barriers to physical activity have been reported, such as nausea [12, 17, 19], feeling tired [18, 19], musculoskeletal pain [12, 18], uterine contractions [12], and fear of miscarriage [15, 18, 19]. Incontinence and discomfort [15, 19] are more common reasons for avoiding physical activity in late pregnancy. Addressing these barriers with tailored information and guidance should be part of routine antenatal care.

Besides pregnancy specific symptoms, lack of time has been given as the most important barrier to physical activity in pregnancy [18, 20]. Since opportunities to increase time spent on physical activity is limited, integrating biking or walking into the daily travel to and from work might be one way to overcome this.

The present study highlights active transportation to school or work as an important strategy to prevent excessive weight-gain in pregnancy. In the global action plan for the prevention and control of noncommunicable diseases 2013–2020 the World Health Organization advice member states “to promote physical activity through activity of daily living” and “to target supportive infrastructure for walking and cycling” [10]. Greater emphasis on public policies which facilitate active transportation might have significant public health benefits including preventing population weight gain [51]. This might be done through an integrated culturally sensitive approach. We suggest an increased emphasis on the benefits of being physically active in antenatal care. Since pregnant women have been found to be more sensitive to barriers than enablers of physical activity [20], it might be wise to emphasis these barriers.

Physical support at work/school such as available bike parking, presence of showers and flexible dress codes [52], as well as cultural and social support for active transportation [52] have been found to be important for women’s transport choices. Further, studies on traditional beliefs connected to pregnancy and lifestyle factors such as physical activity and active transportation in western populations are warranted. Additionally, the importance of changes in infrastructure, such as well-developed public transport, restriction on car use, limited workplace parking and separate bike lines should be addressed.

The significantly higher frequency of premature deliveries in the “active-active” group compared to the “active-less active” group is difficult to explain. The prevalence of preterm birth in the “active-active” group did not differ among women who continue to walk (11 %), bike (11 %) or used public transportation (12 %) in early pregnancy. Large population-based studies have reported physical activity during pregnancy to be associated with a slightly decreased rate of preterm birth [53, 54]. In this context, the present study is small, and the association might be a chance finding. However, preterm birth should be prevented, and this significant association should be further investigated.

### Strengths and limitations

Main strengths are the population-based cohort, the high response rate, few missing data, and the longitudinal design with multiple assessments of body weights. Self-reported weight and height often leads to a systematic underestimation of BMI because weight is commonly under-reported and height often over-reported [55]. Thus, the objective measurement of height and weight during pregnancy in our study is another strength. However, pre-pregnant weight was self-reported. The mean variation between self-reported and measured weight among women has been found to be small [56], but with larger discrepancy in certain groups such as those with large BMI and some ethnic minorities [56, 57]. This might have misclassified overweight and obese women towards normal pre-pregnant weight in baseline measure in the present study, but this bias is probably equal in the active-active and active-less active groups. In the present study, there was no significant difference in pre-pregnancy BMI between the “active-active” and the “active-less active” groups, thus eliminating the bias that lean individuals might be more likely to continue with an active mode of transportation to work or school. However, there was a non-significant difference in pre-pregnant weight between the two groups, where women in the “active-active” group weighted 1.1 kg less than the women in the “active-less active” group. These baseline levels were corrected for in the statistical model. Furthermore, there were no significant differences in baseline diet score, pre-pregnant smoking, educational level, the presence of nausea at inclusion or physical activity level between the two groups. Other limitations were the reliance on self-reported data on mode of transportation to work or school. The data on pre-pregnancy transportation were collected in retrospect, and thus we cannot rule out recall bias. However, the questions regarding transportation showed high levels of test-retest agreement. Furthermore, there was no collection of objective measures of physical activity, exact distance between home and school/work, or time spent on active travel.

The present study examines the effect of *changing to less active* mode of transportation. Several studies have found that people who use public transport to work report a higher level of physical activity than people who use private transport [42, 43]. Thus, public transport was categorized as “active” pre-pregnancy. However, to *change* from walk/bike at pre-pregnancy to public transportation in early pregnancy was categorized as “active-less active”. Because parts of public transportation trips are spent sitting on a bus, train etc., this is naturally less active than walking/biking the full length from home to work. This assumption relies on there being no change of job or home within the study period, data that was not available to us. There is also a potential self-selection bias: women who continue with active transportation in early pregnancy might be more inclined to have a healthier lifestyle throughout pregnancy. Additionally there is potential for substitution bias: women who use active transportation to school or work might decrease their leisure time physical activity because of the extra time they spend on their job journey. However, those who use active transportation in their journey to work have been found to be more likely to reach the recommended levels of daily physical activity [42, 58, 59]. Saelens et al. reported that people who use active transportation to work had higher levels of total physical activity compared to those who did not use active transportation, and that the increased level of physical activity was directly related to an active mode of transportation [42]. The women were assigned to the “active-active” or “active-less active” groups based on only one parameter: change in mode of transportation to work or school. By controlling for factors known to be associated with mode of transportation such as pregnancy complications, educational level, pre-pregnant BMI, smoking, NFFD-diet score and physical activity level we have tried to overcome this. However, we cannot exclude residual confounding. Our results may lack generalizability to other populations because the population in the present study is biased towards older maternal age, and higher educational level [60]. Diagnosed or undiagnosed illness or symptoms related to pregnancy such as heartburn and tiredness might be responsible for both discontinuing active transportation and increased GWG. Unfortunately, we did not systematically monitor illnesses or symptoms related to pregnancy. Women with diabetes mellitus or physical disability which precluded participation in a physical activity program were not included in the trial, limiting reverse causality.

## Conclusions

Women continuing an active mode of transportation to work or school from pre- to early pregnancy had lower GWG than women who change from an active mode of transportation pre-pregnancy to a less active mode of transportation in early pregnancy. This indicates that active transportation is one possible approach to prevent excessive weight gain in pregnancy.

## Ethics, consent and permission

The study has been approved by the Norwegian Regional Committee for Medical Research Ethics South-East C (REK reference 2009/429). The Fit for Delivery trial has the Clinical Trials registration: clinickaltrial.gov NCT01001689. Written informed consent was obtained from all participants.
